# Emission-Responsive Charging of Electric Cars and Carsharing to Improve the Security of Electricity Supply for Switzerland

**DOI:** 10.1021/acs.est.4c13270

**Published:** 2025-07-17

**Authors:** Elliot Romano, Binod Koirala, Martin Rüdisüli, Sven Eggimann

**Affiliations:** † Institute for Environmental Sciences and Department F.-A. Forel for Environmental and Aquatic Sciences, Energy Systems Group, University of Geneva, Geneva 1205, Switzerland; ‡ Urban Energy Systems Laboratory, Swiss Federal Laboratories for Materials Science and Technology, Empa, Dübendorf CH-8600, Switzerland; § Verband Schweizerischer Elektrizitätsunternehmen, Aarau CH-5000, Switzerland; ∥ Department of Geography, 26742 The Hebrew University of Jerusalem, Mount Scopus, Jerusalem 91905, Israel

**Keywords:** carbon intensity, electric vehicle, greenhouse gas emissions, car sharing, smart charging, life cycle analysis

## Abstract

The simultaneous adoption of battery electric vehicles and switching from privately owned cars to carsharing substantially impacts the release of greenhouse gas emissions, mobility costs and the security of electricity supply. An integrated hourly charging behavior optimization model for charging costs or emissions relying on a large carsharing reservation database is showcased for Switzerland, revealing a strong trade-off between electricity prices and CO_2_ emissions. Price-responsive electric charging reduces charging costs by 27% compared to emission-responsive charging, which reduces e-mobility-related CO_2_ emissions by 82%. Introducing a dynamic carbon tariff could make emission-responsive charging economically rational, resulting in an average carbon price of EUR 0.3/kg CO_2_-equivalent. Although carsharing hinders battery charging at times of low emissions and requires increased overnight charging, carsharing only leads to minimal differences in operational costs or charging emissions compared to privately owned cars. However, a large-scale shift to battery electric vehicles requires energy system adjustments to meet the additional electricity needs from e-mobility. For complete electrification of private cars by 2050 in Switzerland, an additional curtailment, storage or import capacity of 1.3 TWh for the most critical winter month is required for individual car ownership and an additional 1.0 TWh for shared e-mobility.

## Graphical abstract

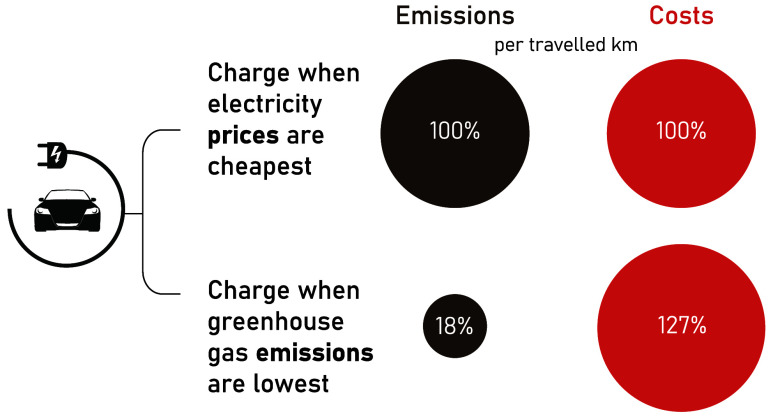

## 1 Introduction

For decarbonising today’s transport system, reducing the number of cars through carsharing and the uptake of battery electric vehicles (BEVs) are two sought-after strategies.^1^ Reducing transport-related greenhouse gas emissions will require the electrification of mobility and a shift in the modal split. Carsharing enables a reduction in the number of cars,^2^ which are shared among multiple users instead of being owned by individual households^3^ and carsharing households are typically more receptive to switching to BEVs.^4^ The total traveled distance per person is affected—among many other factors^5^—by the availability and ownership model of cars.^6,7^ Literature suggests that carsharing can decrease the carbon footprint by 41% if one shared vehicle replaces ten individually owned vehicles^8^ and can lower car travel times to reduce transport-related energy consumption by 25%, as observed in a Swiss case study.^9^ A reduction of the number of cars combined with a travel distance reduction (e.g., due to shifting to other modes of transportation) would not only reduce car-based greenhouse gas emissions but also impact the electricity sector through improving the security of electricity supply, which is of concern in the case of large-scale BEV uptake.^10^ Electrification through BEVs enables smart battery charging strategies at an individual or aggregated level^11^ to reduce costs or emissions from electric charging. However, carsharing affects how vehicles can be charged and the ability of batteries to supply power back to the grid.^1^ Given the more frequent and intensive use of shared vehicles, BEVs may face challenges in implementing smart charging due to lower charging flexibility throughout the day, potentially mitigating environmental carsharing advantages. Furthermore, the large-scale uptake of BEVs and pursuing carsharing affect the electricity demand within a country that must be satisfied.

### 1.1 BEV Sustainability Assessment

The sustainability of internal combustion cars versus BEVs has been studied through life-cycle assessments.^12,13^ The overall emission footprint depends not only on the distance traveled, calendar aging or driving intensity, but also on operational emissions from charging.^14^ Typically, temporal differences in embedded greenhouse gas emissions in electricity for operating vehicles are ignored.^15^ Instead, fixed emissions factors are assumed.^2^ However, emission factors are closely intertwined with a country’s electricity consumption mix,^16^ where, depending on the time of electric charging, the uptake of BEVs may increase overall emissions compared to diesel or petrol cars.^17^ Therefore, accounting for the CO_2_ content of electricity and relying on low-carbon electricity is essential. For example, for China, the uptake of BEVs was simulated to shift gasoline consumption to coal-fired power generation, thereby causing higher overall CO_2_ emissions.^18^ Similar observations were made for other contexts, such as in California^19^ or Germany.^20^ Different charging strategies revealed that overnight charging could facilitate a 12–20% increase in BEV penetration in Switzerland^21^ or through coordinated charging, simulations for a California case study show annual CO_2_ emission reductions of 18% and peak power reduction of 33%.^22^

### 1.2 Price- versus Emission-Responsive Charging

BEV users typically show battery charging behavior to save costs, known as *price-responsive charging*.^23^ To minimize charging costs, users often take advantage of variable electricity prices. Various dynamic pricing schemes exist, ranging from real-time pricing—e.g. updated hourly—to simpler time-of-use tariffs, which apply different rates over broader time blocks, such as day versus night or weekdays versus weekends.^24^ Price-responsive charging not only reduces electricity expenses but also promotes more efficient grid utilization by helping to balance electricity supply and demand. In this paper, price-responsive charging is assumed to be enabled by real-time electricity pricing,^25^ which allows users to fully benefit from the hourly variability of electricity prices throughout the day.^24^ Smart grids, equipped with real-time data meters or devices making available price forecasts, can inform consumers about electricity prices while additionally providing information on the environmental impacts of electricity consumption in terms of CO_2_ emissions. This latter information enables *emission-responsive charging* ^26^ as integrating CO_2_ intensity information into smart meters allows consumers to make environmentally conscious decisions about when to charge by aligning charging preferences with periods of lower embedded CO_2_ emissions. In the case of emission-responsive charging, the use of low-emission electricity generation technologies (i.e., hydro-power, solar and wind) is maximized, especially when these technologies–dispatched at low cost–are abundantly available in the supply mix. Furthermore, particularly imported electricity from coal-fired power plants is minimized. Emission-responsive charging thus facilitates the integration of renewable technologies. Price or emission-responsive smart charging can help to reduce generation curtailment and be performed for both privately owned and shared vehicles, which influences how and when vehicles are used.

### 1.3 Aim, Scope and Structure

Here, a combined assessment of BEV use considering different charging behaviors under distinct car ownership models is presented. This paper is structured in two main parts: First (Sections 2.1–2.3 and 3.1–3.4), the focus is on charging strategies and exploring costs and CO_2_ emissions from charging, without considering the absolute number of privately or commonly owned vehicles in Switzerland. Second (Sections 2.4 and 3.4), the total number of vehicles is explicitly modeled, quantifying electricity supply implications in the case of large-scale vehicle uptake at a national scale.

## 2 Materials and Methods

An overview of the main methodological steps and the data sets used is shown in Figure 1.

**Figure 1:**
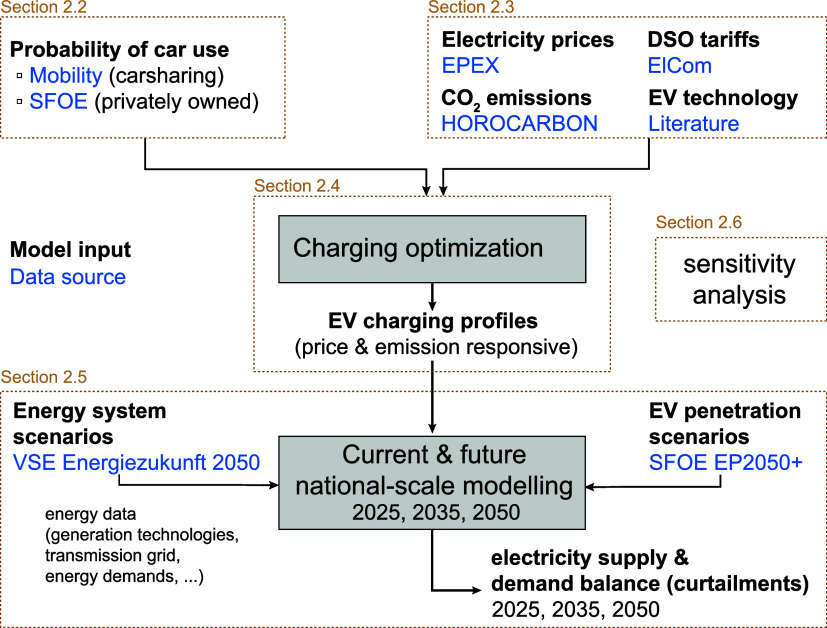
Schematic methodological overview and used data sets shown in blue.

### 2.1 Swiss Case Study Background

Switzerland, as the “motherland of car-sharing”,^6^ is selected as a case study due to the availability of car reservation data, high-resolution electricity emission factors and electricity prices. This data availability enables a holistic quantification of cost, CO_2_ emissions and electricity curtailments considering charging behaviors and car ownership models. Switzerland is a net electricity producer but strongly depends on electricity imports from neighboring countries, particularly during winter months, causing concerns about future large-scale BEV uptake.^10^ Switzerland’s imported electricity mix comprises both renewable and fossil fuel-based generation, which influences the CO_2_ footprint of the electricity consumed. The total CO_2_ emissions produced by the Swiss transport sector decreased from 16.3 Mt of CO_2_ in 2012 to 13.7 Mt in 2021,^27^ which coincides with the evolution of BEVs and the installation of charging stations. The ongoing electrification of mobility is rapid, with BEVs making up over 35% of new vehicles,^28^ but is recently increasingly in competition with hybrid vehicles.^29^ Remarkably, the uptake of BEVs has exceeded targets with no direct national purchase incentives and only modest state support. However, the slow deployment rate of smart meter infrastructure, with currently only ∼ 30% of all households having smart meters,^30^ and the lack of data information platforms providing real-time information to car owners or fleet operators limits smart charging capabilities and hinders BEV users from making informed decisions about when to charge. Furthermore, as of today, full dynamic price or environmental signals at high temporal resolution are not yet commonly available in Switzerland for either private households or mobility fleet operators.

### 2.2 Probability of Car Use

A fleet of BEVs for Switzerland is modeled, whereby cars are assumed to be parked at the same location and driven during specific times and days. BEVs are operated by two schemes: for the *individual car ownership* scheme, cars are owned by private users. For the *carsharing* scheme, cars are shared among multiple users. Carsharing is assumed to be adopted to substitute for the entire private vehicle fleet, and a profile of use is determined by focusing on urban areas where carsharing is adopted most prominently. For the individual car ownership scheme, short-term driving patterns are largely predictable due to the fixed working hours, fixed business schedules and identical routes. Individual car ownership driving patterns and the probability of charging are derived from clustering survey data from a representative sample of inhabitants.^31^ To calculate the hourly distribution of the probability of driving for the carsharing scheme, typical car usage and driving patterns are derived from real-world data from fixed carsharing stations (as opposed to free-floating)^32^ provided by the largest Swiss shared mobility provider^33^ as follows: From a data set containing around 1.5 million Swiss car reservations for 2021, cars located within major cities and of certain types (Economy, Budget, Combi, Micro) are filtered and reservation time patterns are aggregated throughout the year. The resulting probability of car use (or reservation) over the weekdays, and hour is classified uniformly within five classes, whereby effects from holidays are ignored (Figure 2). As such, activity chains and user behavior are not explicitly modeled, compared to, for example, agent-based modeling approaches.^34–36^ The probability of driving is used as an exogenous model input in the optimization approach and acts as a constraint on how and when charging is considered feasible. Whereas both schemes are modeled for the entire private fleet, in the real world, a mix of both schemes would be found, with modeling results somewhere between the two conceptualised extremes.

**Figure 2:**
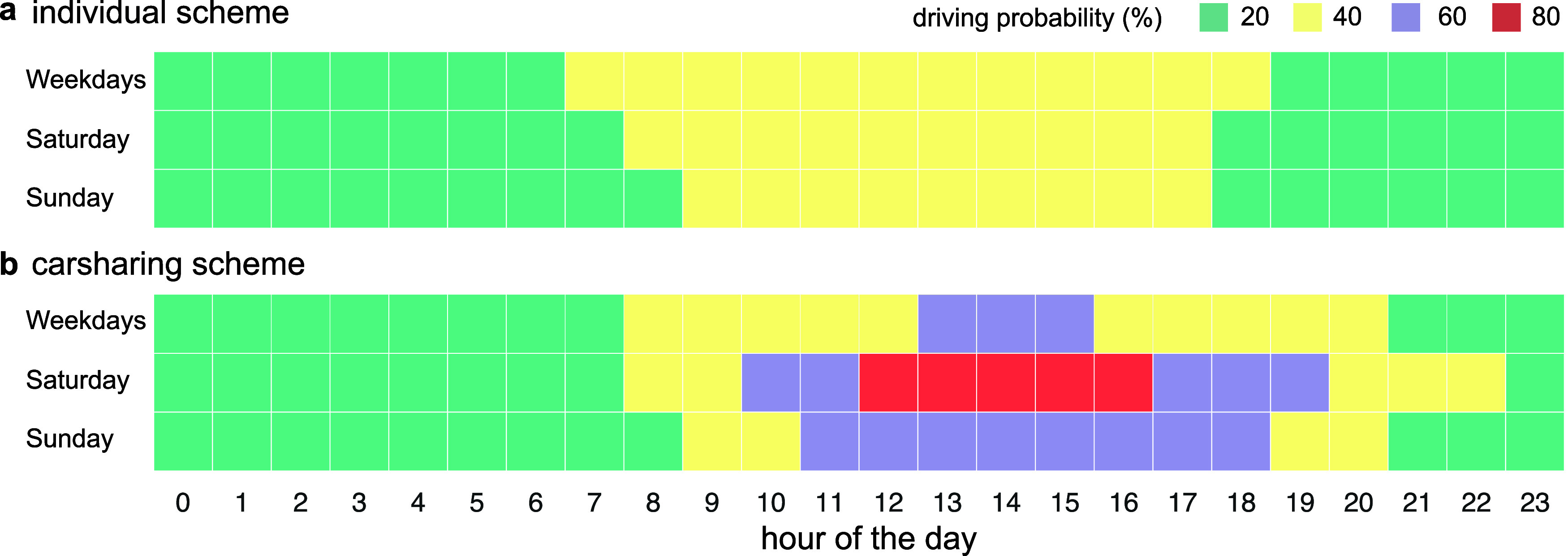
Probability of car use for every hour in a day and different types of days. The probability of car use is calculated for weekdays, Saturdays, and Sundays for the (a) individual ownership scheme based on literature and (b) the carsharing scheme using actual car reservation data in Swiss cities for 2021. Values are always rounded to the upper maximum class value.

### 2.3 Greenhouse Gas Emission and Price Data

Greenhouse gas emission values are taken from HOROCARBON,^37^ which employs a methodology that calculates greenhouse gas intensities of consumed electricity in grams of CO_2_-equivalent per kilowatt-hour (g CO2eq/kWh), accounting for imports and exports with neighboring countries.^38^ This calculation determines an emission factor at an hourly time scale as the ratio of absolute emissions from domestically produced and imported electricity to the volume of consumed electricity. Charging is assumed to occur through electricity purchases on the spot market,^39^ even though car owners or companies providing carsharing may be billed at different tariff schemes or prices, as they do not have direct market access. An additional fee of €0.13 kWh is included for grid distribution, which reflects the median electricity tariffs for grid usage by households as billed by Swiss distribution system operators, regulated by the Swiss energy regulator (ElCom). These feeds do not account for potential future cost increases, particularly those arising from higher capacity charges associated with the expansion of fast-charging infrastructure. Costs related to charging infrastructure—such as those depending on charging type, location, grid reinforcement, or grid interaction—are not considered.

### 2.4 Optimization of Charging Schedules

Whenever a vehicle is driven, charging is not feasible. To plan charging and discharging at the aggregated level, representative driving patterns and the probability of car use is considered for the entire vehicle fleet. A charging schedule is simulated to guarantee that all required trips, distances, and additional constraints are met. BEV owners are assumed to make informed charging decisions that align with their preferences to minimize electricity costs or reduce environmental impact. The charging schedule must ensure that vehicles can cover minimum weekly distances while considering battery capacity, CO_2_ emissions and charging costs. Furthermore, vehicles must be charged to fulfill a minimum state of charge before use. Charging behavior is modeled using a Mixed-Integer Linear Programming (MILP) approach, rooted in rational decision-making and assuming perfect foresight of electricity prices and CO_2_ emissions. The Pyomo package and GNU Linear Programming Kit are used as a solver. The objective function minimizes either monthly costs or the CO_2_ footprint from charging. For each month, the objective function considers all hours within a month. The BEV driving patterns are characterized by their hourly discharge rate. If a vehicle is likely to be driven during a given hourly interval with a certain probability, the maximum electricity that can be recharged in that interval is weighted by the probability that the vehicle will not be driven during that hour. The objective functions for costs (eq 1) and environmental impact (eq 2) are formulated as a minimization problem for the entire vehicle fleet subject to different constraints. Several variables and constraints are introduced to account for the battery state of charge, driving patterns, and charging behaviors of BEV users.

1 $$\min\;\mathrm{COST}=\int_{0}^{T}L\left(t\right)P\left(t\right)dt$$

2 $$\min\;\mathrm{EMISSION}=\int_{0}^{T}L\left(t\right)E\left(t\right)dt$$

3 $$SoC\left(t+1\right)B_{\max}=SoC\left(t\right)\times B_{\max}-D\left(t\right)\times S\times \eta +L\left(t\right)\times C$$

4 $$B_{\min}\leq \mathrm{SoC}\left(t\right)B_{\max}\leq B_{\max}$$

5 L(t)≤1−D(t)

$$\overset{T}{\underset{t=t_{0}}{\sum }}D\left(t\right)\times S\geq \mathrm{TO}T_{\mathrm{km}}$$ 6

where *t* ∈ [0, *T*] is the hourly period over a month interval, *B*_max_ is the maximum battery capacity (kWh), *L* is the charging decision variable, *E* is the CO_2_ emission factor of electricity (g CO2eq/kWh), and *P*(*t*) is the hourly electricity price (€/kWh) at time *t*. The first constraint (eq 3) determines the state of charge (SoC) in relation to the SoC of the previous time step. At time *t* + 1, the SoC depends on the electric vehicle’s charged energy (kWh) and energy demand (kWh) at the time step *t*. *S* is the average distance driven in an hour, η is the energy demand (kWh) per 100 km, and *C* is the charging rate in kWh per hour. The second constraint (eq 4) restricts the battery’s SoC within a defined minimal and maximal range (*B*_min_, *B*_max_). The minimum battery charging state is the minimum acceptable reserve, assumed as 15% and expressed as a percentage of the maximal value *B*_max_. Equation 5 ensures battery charging only when vehicles are parked, *D* being the probability of driving at period *t* and, therefore, the charging decision *L*(*t*) is a function of the probability of being available for charging. As such, the motivation behind trips or travelers’ activity chains are not explicitly modeled but implicitly included in the real-world data set used to derive *D*. The battery degradation is not explicitly modeled, and no distinction is made between degradation rates for individually owned and shared vehicles, which may affect overall environmental performance. The electricity demand is modeled based on the assumed minimum distance driven per year (eq 6), which is evenly distributed across each month and week. Here, a simplified approach is pursued by relying on average travel speed (*S*) and distance to estimate electric energy demand, while not explicitly specifying different vehicle types. Instead, a typical electric vehicle battery capacity of 70 kWh is assumed, corresponding to the standard battery capacity in currently available BEV models. Alternatively, high-fidelity physics-based modeling approaches^40,41^ could be implemented for detailed energy consumption calculation, if explicit route information and vehicle type distribution information are available. Given the scope of the presented analysis and data availability, the electric energy demand estimation does not explore further the sensitivity of vehicle velocity or routing characteristics (e.g., slope) or regenerative braking. To obtain comparable results between the individual ownership and carsharing regime, cost and emissions are calculated for a total average traveled distance per car (TOT_km_) of 14,000 km per year for privately owned and shared vehicles, corresponding to the traveled average distance per car in Switzerland.^42^ The assumed traveled distance per car considerably impacts the overall optimization and needs to be carefully motivated (cf. Section 2.5). The distances traveled by shared cars thereby may depend on the carsharing intensity, the length of the average trips, or the interplay with other modes of transport. An energy demand of 0.2 kWh per km (η) at an average speed of 15 km/h (*S*) is assumed for both regimes, corresponding to an hourly average energy consumption of 3 kWh. The average speed assumptions are considered to provide realistic travel characteristics for the Swiss urban case study setting. They were derived from the underlying carsharing reservation data set by dividing the total driven kilometres by the total reservation time. This estimate is in good accordance with speed assumptions by others.^43^ The power of the charging infrastructure (*C*) is assumed to be 11 kW, which is considered here to be a typical charging infrastructure for individual users at home and at work. No detailed modeling was performed for the battery, i.e. the round-trip efficiency of batteries and other types of battery charging losses are ignored, which, however, may be considerable and can exceed 10%.^44,45^ The sensitivity of the analysis to faster charging for shared mobility users is explored in Section 2.5. In the optimization, the charging power is implemented so that power is reduced depending on the probability of the car being driven, i.e., the charging rate is multiplied by one minus the probability of car use. The optimization is run for 2017–2021, and average annual results are reported. Even though the cost and emissions data include the COVID year 2020, the probability of car use is based on the year 2021 with more representative behavior patterns.

### 2.5 National-Scale Modeling

Implications of BEV users minimizing costs or CO_2_ emissions are estimated at the national scale for an entire e-mobility fleet. For Switzerland, a fleet of around 3.6 million BEVs is predicted by 2050, corresponding to an estimated 13 TWh of additional annual electricity demand,^46^ corresponding to approximately 21.1% of the total current electricity demand. To model the future energy mix for Switzerland, an hourly European-scale electricity demand and supply balance model was developed in Antares.^47^

For European countries, the assumptions on the increase of the electricity load through electrification of thermal demand and mobility for the residential sector alongside additional generation capacities of solar photovoltaics and wind, battery storage capacity, increased utilization of hydro capacities and progressive phase-out of nuclear power plants are taken from the TYNDP 2020 Global Ambition scenario.^48^ Specific data for Switzerland regarding electricity demand, available import capacities and generation profiles of solar photovoltaic are modeled according to the “Swiss Energy Perspectives 2050+” report.^49^ A heuristic dispatch approach is applied for flexible hydropower generation (dam, pumped-hydro storage). Hydrogen and synthetic fuels are assumed to complement the electrification of heavy trucks and special vehicles, but are not explicitly modeled. The simulated growth of BEVs in the fleet of personal cars is assumed to evolve from 28% by 2025 to 60% by 2030 to a complete electrification scenario by 2050, corresponding to an estimated additional 13 TWh^46^ per year of electricity by 2050. For the carsharing scheme, a reduction of 25% of the anticipated total number of EVs is assumed for each time step (2.2 million by 2030, 3.6 million by 2050). The reduction in energy demand from carsharing is set to be proportional to the reduction of cars, as the total observed traveled distance per shared vehicle was similar to the distance traveled by private cars in our data set. Notably, this assumption is critical for quantifying future changes to simulated electricity demand, and the exact savings will differ depending on the context. Integrating future BEV electricity demand in a static current power system falls short of accounting for changes in evolving distribution grid capacity expansion or the dynamic interplay with future prices. A simplified approach is pursued to model potential future energy demand and curtailment by relying on the same charging probabilities of today, which are, however, adapted to the energy needs of the future car fleet. Even though future prices and emissions might change the probability of charging and uncertainties for such long-term projections are high, the posed modeling approach provides insights into what-if scenarios for limited energy system evolution or limited grid expansion investment. Independent of when the future load (i.e., charging) will occur, at the monthly scale, periods of insufficient energy supply will require curtailments, i.e., a reduction of the services that require electricity.

### 2.6 Sensitivity Analysis

A local sensitivity analysis is performed to explore the impact of the charging rate assumption and the difference in the traveled distance of cars from the individual or carsharing scheme (see results in Table 2). In the case of higher charging rates (i.e., fast charging), the potential to optimize for emissions and costs is expected to be higher, as charging needs to occur less frequently in times of high costs or emissions. Three rates for regular (11 kW), fast (22 kW) and very fast (55 kW) charging are explored. Whereas higher charging rates are explored for shared mobility, only regular charging is considered for the individual ownership model. As shared mobility cars can typically be assumed to be used more intensively, we increase the traveled distance by 25%, 50%, 75% and 100%. Whereas absolute costs and emissions per traveled km are directly linked to the assumed traveled distance, this sensitivity allows for assessing the impact on the potential of responsive charging (for cost and emissions) for both schemes.

## 3 Results

### 3.1 Probability of Charging and Car-Ownership Scheme

Distinct driving times and probability of car use are observed for different day types (weekday, Saturday and Sunday) for price- and emission-responsive charging (Figure 2). Privately owned cars typically sit idle for most of the time.^50^ Actual car reservation data reveals that shared vehicles are driven more during mid-day hours, especially on weekends.

### 3.2 Low Emissions When Electricity Prices are High in Switzerland

A broadly consistent negative relationship is observed between electricity prices and CO_2_ emissions, with lower emissions in times of higher prices and vice versa (Figure 3). The highest absolute prices and emission values occur during the winter months, and the lowest emission factors and prices occur during summer. This observed pattern of lower emission values in times of higher electricity prices is mainly explained by Switzerland generating and exporting electricity from hydro dams—which have low CO_2_ factors—when electricity prices are high. Conversely, Switzerland imports electricity from fossil-fuelled power plants from neighboring countries during times of low electricity prices. Even though the emission patterns arising from the Swiss electricity generation mix and the ability to charge and discharge pumped hydro and hydro reservoirs depending on market prices are distinct, the trade-off between electricity prices and CO_2_ emissions has been noted more generally,^17–19^ and similar electricity footprint trade-offs can be observed elsewhere, such as in California^17^ or Australia.^51^

**Figure 3:**
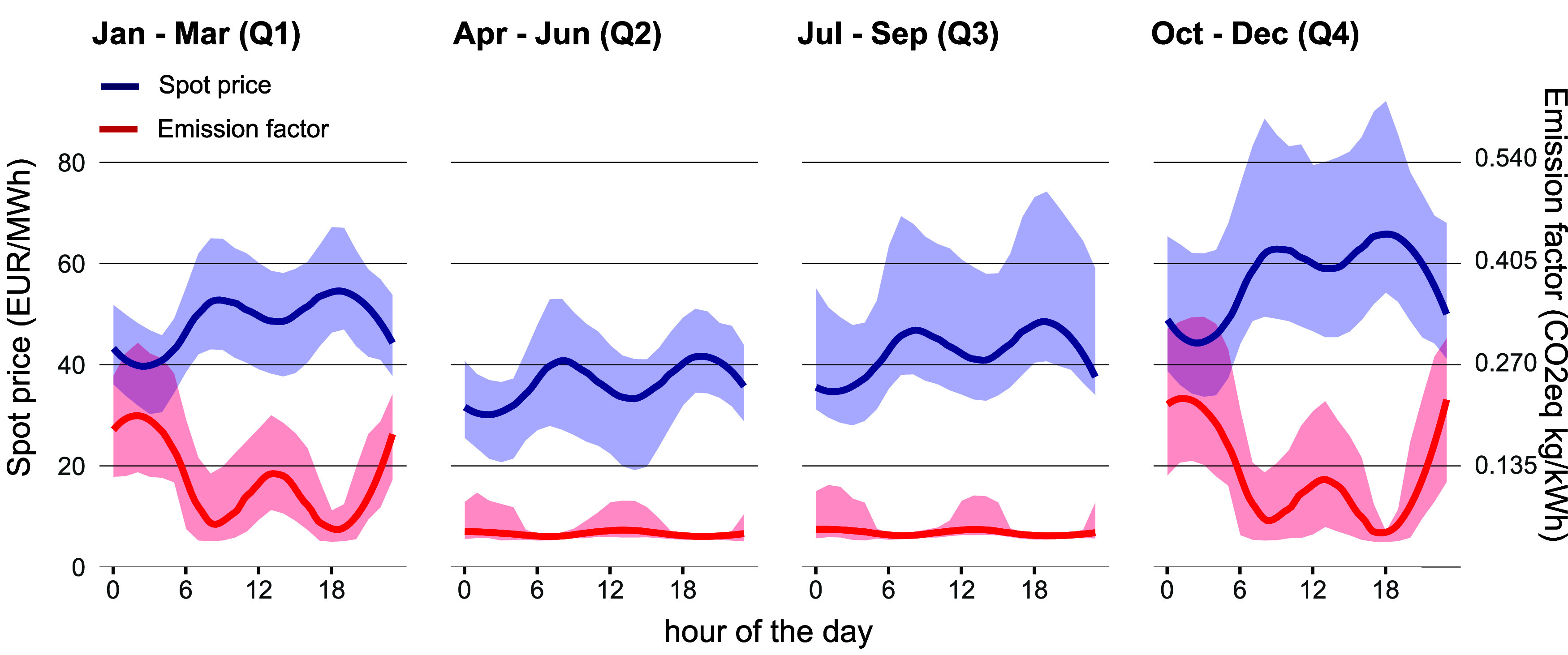
Seasonal electricity costs and CO_2_ content in Switzerland for the years 2017–2021. Generally, an inverse relationship is observed between the emission factor in kg CO2eq/kWh of consumed electricity (right axis, blue line) and the average spot prices (€/MWh) (left axis, red line). The relationship is less consistent for Q2 and Q3 due to higher availability of renewable generation (mostly hydro) and lower imports. The overall pattern is similar across the different years. Bold lines represent average seasonal values, and the shaded area represents the 25–75% confidence interval.

### 3.3 Electricity Load Impacts of Smart Charging and Shared Mobility

At which point in time a vehicle is charged depends not only on whether the vehicle is being driven or whether electric charging infrastructure is available. The time of charge can also be an explicit decision of users, based on to their price- or emission preferences. The simulated charging probabilities through a linear optimization process considering emission- or price-responsive behavior and different car-ownership schemes reveal when batteries are recharged throughout the year and within the day (Figure 4). The time at which electricity is consumed shows distinct patterns between user behavior, seasons and ownership schemes. For price-responsive charging, peak load is observed at dawn between 4–6 (individual scheme) or, more generally, within the night hours. Meanwhile, a second peak is observed for the individual scheme in the afternoon, especially in summer, when prices drop due to excess renewables. For carsharing, the afternoon charging peak is less pronounced. Overall, the probability of charging at night under the price-responsive regime increases for the carsharing ownership scheme from 56% to 66%, as cars are driven more during day hours (8–20), thus decreasing the feasibility of charging at times of low prices when high shares of renewables are available.

For emission-responsive charging, the peak load is observed later in the morning (8–10), with a second peak in the evening hours (6–9) in winter (October to March). This pattern holds for both ownership schemes, whereas most of the charging occurs up to 10 am, with only a small amount of charging in the afternoon in summer (April to September). During those hours, the carshared fleet is not entirely driven, which thus allows car batteries to be partially charged. The probability of charging at night under the emission-responsive regime falls to 35% and is almost similar for both ownership schemes. The monthly distribution of prices and emission values is shown in Figure 5 and Table 1.

**Figure 4:**
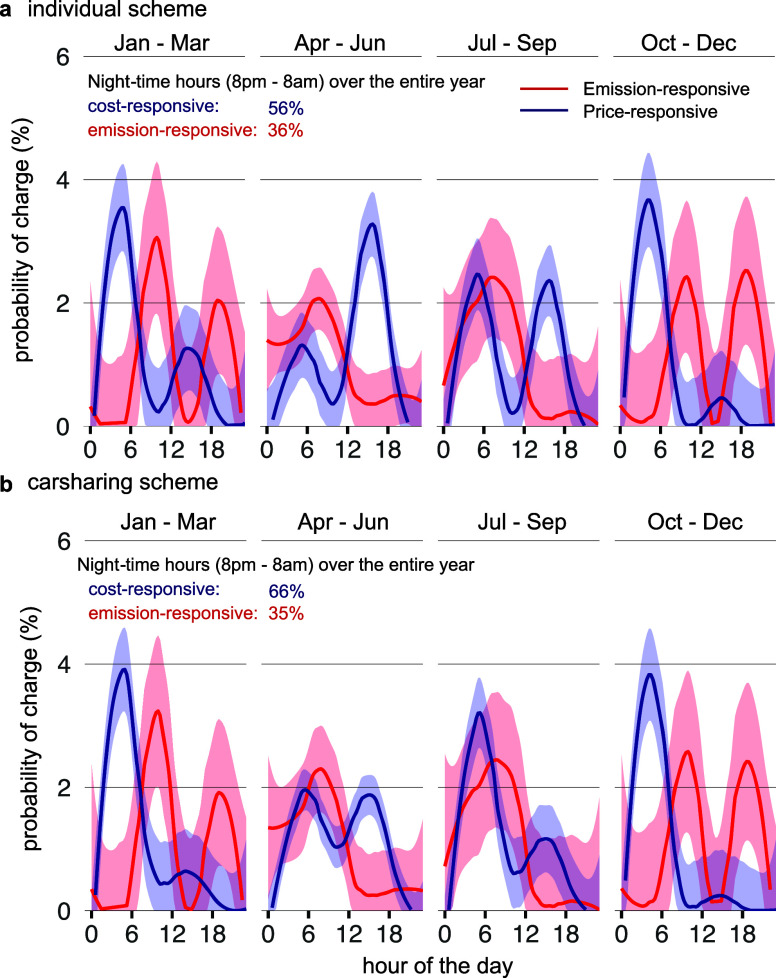
Comparison of charging for price- and emission-responsive behavior based on the years 2017–2021. Simulation results are shown for (a) individual car ownership and (b) carsharing. The probabilities are averaged across all days of the simulated years and the shaded areas show the 95% confidence interval.

### 3.4 Economic and Environmental Benefits from Smart Charging or Carsharing

Aggregated results over the entire year reveal, on average, higher prices when optimizing for emissions (€ 0.034/km, 5 g CO_2_/km or 2g CO_2_/kWh) and, on average, higher emissions when optimizing for costs (€ 0.027/km, 27 g CO_2_/km or 135 g CO_2_/kWh). Depending on the charging scheme, potential cost- or emission savings are considerable: an increase of 27% in costs is simulated for emission-responsive charging and a saving of 81.4% in CO_2_ emissions for emission-responsive charging. Particularly in winter, charging can occur during hours with very high CO_2_ intensities of up to approximately 42 g CO_2_/km (or 210 g CO_2_/kWh) (Figure 5). Analogously, when optimizing for reducing emissions, charging can occur in hours with very high electricity prices of up to € 0.048/km. Overall, these savings are higher than the ones reported in other studies. For example, optimizing charging electric buses for a German city reduced costs by ∼ 14% and CO_2_ emissions by ∼ 15%.^52^ The higher values might be explained by the high reliance of the Swiss electricity system on imports from abroad, combined with a high share of low-carbon electricity in the Swiss electricity mix. Crucially, the differences in overall costs and emissions between private car ownership and carsharing are minimal. Only minor differences are observed, even though, due to the higher probability of car use during the day for the carsharing scheme, it becomes less feasible to take advantage of low-cost hours occurring at midday, especially in summer.

**Figure 5:**
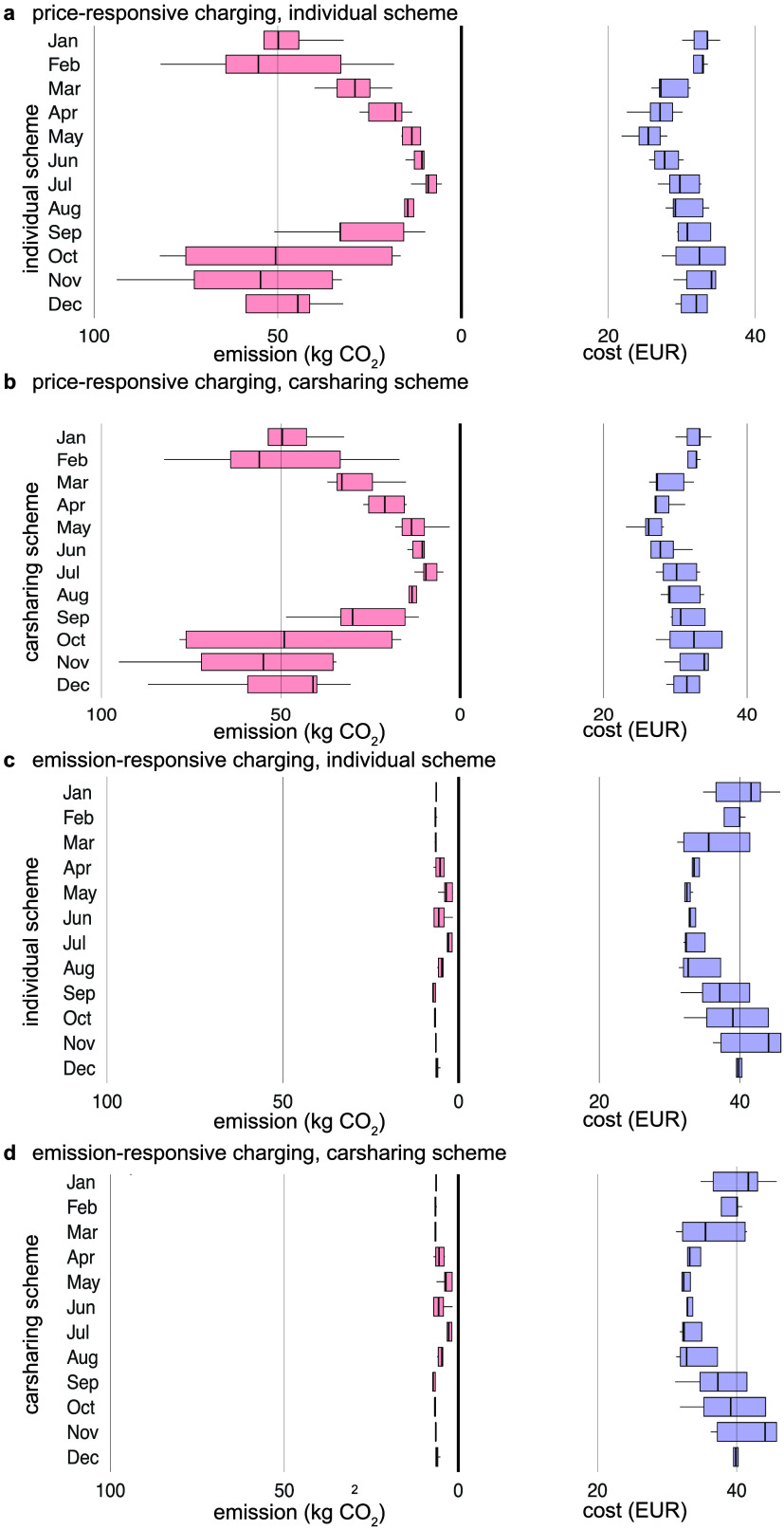
Comparison of total monthly CO_2_eq emissions and costs for price- (a, b) and emission-responsive charging (c, d) and between the individual (a, c) and carsharing scheme (b, d). Boxplots are created with values for the simulated years 2017–2021. Outliers are not shown in the boxplots.

**Table 1:** Overview of Average Calculated Values Per Km Driven for Price- or Emission-Responsive Charging and for the Individual and Carsharing Ownership Scheme Based on the Years 2017–2021

	individual scheme		carsharing scheme		Δ (difference in %)	
	cost^a^	emission^b^	cost^a^	emission^b^	cost^a^	emission^b^
**Price-Responsive Charging**						
average (per km driven)	2.69	27.81	2.71	27.62	+1.1	–0.1
minimum (per km driven)	2.36	18.34	2.53	17.95		
maximum (per km driven)	3.27	41.95	3.33	42.21		
**Emission-Responsive Charging**						
average (per km driven)	3.41	5.06	3.42	5.07	+0.3	0.2
minimum (per km driven)	2.88	4.24	2.89	4.35		
maximum (per km driven)	4.81	6.31	4.84	6.36		
**Δ (Difference in %)**						
average	+27	–81.9	+25.9	–81.7		

a Average costs in EUR cent per km.

b Average emission factor in g CO_2_eq/km.

Several findings can be drawn from the sensitivity analysis, which explores different charging rates and traveled distances (see summary of sensitivity analysis in Table 2 and probability of charge in the Supporting Information): with faster charging, the flexibility of timing to charge increases as the minimum charging state can be reached much faster. In the case of very fast charging, the optimization (i.e., comparison between individual and shared mobility ownership model) improves by up to 3–8% for costs and 8–24% for emissions. Compared to regular charging, in the case of price-responsive charging, with fast charging, more charging occurs in the afternoon, particularly from April to September. For emission-responsive charging, higher charging rates reduce the charging probability in the morning hours in winter. However, the overall charging profiles are similar. For seasonal imbalances (i.e., security of supply shortages), the charging rate has no real effect.

**Table 2:** Cost and Emission Savings (as a Percentage) of the Shared Mobility Scheme Compared to the Individual Scheme^a^

		travel distance increase of shared mobility scheme compared to individual scheme (%)						
		0		+25	+50	+75		+100
**cost differences (%)**								
charging rate	regular (11 kW)	0.8		27.5	54.5	81.7		109.1
	fast (22 kW)	–1.4		24.9	51.4	78.1		105.1
	very fast (55 kW)	–3.3		22.5	48.7	75.2		101.8
**emission differences (%)**								
charging rate	regular (11 kW)		–0.2	28.1	57.4	87.4	118.4	
	fast (22 kW)		–5.6	20.8	47.9	75.6	103.9	
	very fast (55 kW)		–8.8	16.0	41.5	67.8	94.6	

a For the individual scheme, regular charging is assumed in all calculations. The table depicts the ratio ((shared/individual) × 100) between the simulated scenario (carsharing) and the reference scenario (individual, same travel distance and low-charging).

The assumed travel distance for the shared mobility scheme considerably impacts the overall optimization and needs to be carefully chosen. With increasing traveled distance, costs and emissions increase (see Table 2), with the increase being disproportionate, yet still within the same general order of magnitude. Furthermore, the loading profile is generally similar (see SI Figure 2).

### 3.5 Security of Electricity Supply Impacts

Figure 6 depicts the Swiss monthly aggregated electricity demand and generation mix in the case of large-scale BEV uptake. The limited availability of low-carbon electricity, typically from solar or wind, may constrain the emission-responsive charging potential, particularly during winter. Considering total Swiss electricity demand, the difference between the individual ownership and carsharing scheme is small, as in absolute terms, electricity demand from private mobility is 16.8% of the total electricity demand by 2050.

**Figure 6:**
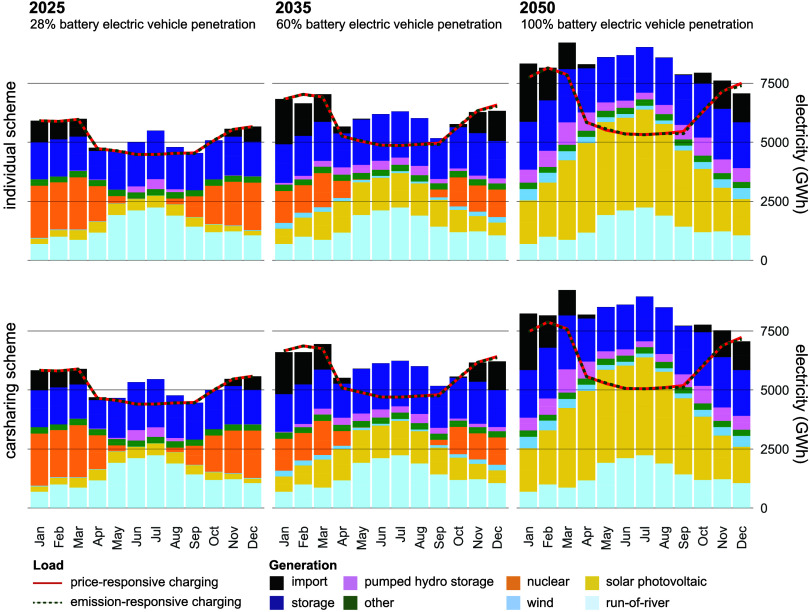
Forecast of monthly electricity demand and generation for Switzerland for the years 2025, 2035, and 2050. Electric demand for BEVs is included, considering vehicle penetration as a percentage of the total private vehicle fleet, corresponding to 13 TWh/y for the individual ownership regime with price- and emissions-responsive charging. The difference between the generation and required load is covered through imports, which are highest during the winter months.

Figure 7 shows monthly levels of unsupplied electricity for different BEV penetration levels from 2025 to 2050, considering anticipated available generation and import capacities for Switzerland. In summer, enough electricity is available to satisfy personal vehicle electrification. Yet, by 2035 and 2050, load shedding (or additional electricity imports) would be needed in the winter, for both price- and emission-responsive charging and for both car ownership schemes. The highest curtailments of electricity demand are observed with the price-responsive regime, requiring a cutoff of up to 12.9% (909 GWh) of total monthly electricity demand for February 2035 and 17.2% (1289 GWh) for December 2050 with the private ownership (individual) scheme. The curtailments are slightly higher for price-responsive charging compared to emission-responsive charging. One reason is due to transmission import constraints with neighboring countries, which occur overnight when price-responsive users charge their vehicles. Furthermore, hydro-generation is not shifted to night hours during winter months, as it still needs to fulfill the day-demand. Our simulation reveals that mobility demand cannot be satisfied in these specific winter months. Even with carsharing, monthly curtailments rise to 10.8% (744 GWh) by February 2035 and a maximum of 14.2% (1026 GWh) by 2050 for the price-responsive scheme. For the emission-responsive scheme, load curtailment is limited to 408 GWh (private ownership) and 323 GWh (carsharing) by 2035. The maximum curtailment for emission-responsive in carsharing mode is 995 GWh in December 2050, as critical supply conditions are still expected to occur during winter. Therefore, neither emission- nor price-responsive charging combined with carsharing ownership will be sufficient to allow for the full electrification of the personal car fleet with monthly unsupplied electricity peaking at between 1.0–1.3 TWh, depending on the charging behavior and ownership regime. In winter, Switzerland already today relies on electricity imports from neighboring countries and without a combination of additional capacity expansion, energy savings or infrastructure updates such as seasonal storage, electrifying the entire personal car fleet will cause challenges that must be addressed.

**Figure 7:**
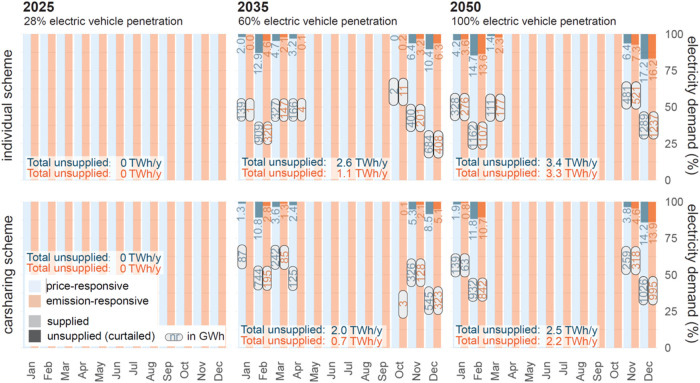
Swiss electricity demand for different BEV penetration scenarios, charging behavior and car-ownership schemes for the years 2025, 2030, and 2050. The share of supplied and unsupplied electricity demand is calculated on an hourly basis and aggregated per month and year. The numbers at the top provide the percentage of curtailed electricity as a percentage of total demand; values within boxes represent the curtailed absolute electricity demand in GWh.

## 4 Discussion

### 4.1 Modeling Limitations and Future Research Needs

The presented modeling approach is based on several limitations and simplifications that can affect the results, and the model assumptions that reflect how future trends in mobility will manifest themselves must be carefully evaluated. The driving patterns are taken as an independent input derived from real-world data, and the focus was on optimal charging strategies, and not on modeling traffic dynamics, charging capacities, or how users decide to drive their cars or optimize trips. The employed linear optimization model assumes perfect rationality, and the modeling approach ignores the capacity limitations of the charging infrastructure.^53^ Cost and emission values, therefore, represent best-case scenarios using perfect foresight on emission or price signals. In reality, BEV users may be unable to fully adapt their schedule according to optimal charging. To model shared mobility use behavior, a real-world data set reflecting current shared mobility patterns in Swiss urban areas is used. Even though the used data set is comprehensive, it still reflects a system where the individually owned vehicle scheme dominates. Particularly in the case of large diffusion of carsharing, car usage patterns may differ. Furthermore, vehicle-to-grid charging or implications of fast charging were not considered in more detail besides the provided sensitivity analysis (see Section 2.5), which could considerably change the electricity supply profile to enable demand-side management and short-term storage.^54^ However, our sensitivity analysis indicates that by having more fast-charging opportunities, the potential for minimizing emissions or costs is amplified. Nevertheless, the challenges of seasonal storage and winter electricity shortages due to the low availability of renewables and high electricity demand remain.

This study only considered passenger cars, and considerations for commercial vehicles such as buses and lorries are not included. A particular challenge of modeling a fleet of BEVs is that the electricity generation mix and CO_2_ intensity of electricity imports may change due to the additional electricity demand, requiring dynamic energy system modeling incorporating endogenous links between BEV charging electricity demand, price or emission signals as well as an in-depth understanding of the price elasticity of BEV charging demand. More generally, future costs and the temporal pattern of embedded emissions of electricity may vary and impact the simulation results of the presented analysis. Such considerations are beyond the scope here but pose future refinement opportunities. A key contribution of this work is the temporally explicit analysis of operational emissions, and it is essential to combine the findings presented here with life-cycle analysis that also include embodied emissions. Further investigation could also assess other benefits from controlled charging strategies, such as improved integration of renewables with associated lower CO_2_.^18,55^ Besides the focus on cost and emissions, aspects of comfort, usability, or cars as status symbols could be explored further, all aspects that influence evolving mobility trends.^50^ Achieving both a high share of BEV and introducing carsharing is not straightforward and a socio-technical challenge:^56–58^ This means that besides solving engineering challenges, such as ubiquitous charging and interaction with the electricity grid, mobility transitions also require a change in social acceptance, behavior, or business models.^50,59–63^ The success of carsharing is closely tied to the broader availability and quality of alternative transportation modes, as shared vehicles alone cannot meet all mobility needs. Shared mobility works best as part of a multimodal transport system, where public transportation, cycling, or walking options are easily available.^6^ Finally, beyond addressing challenges like increased electricity demand, wider-reaching impacts of BEVs and carsharing can be further explored.

### 4.2 Implications

Sustainability assessments of BEVs must include emissions from electric charging to obtain a full life-cycle assessment and emission balance.^64^ Having readily available price and CO_2_ emission signals allows for implementing smart BEV charging strategies to increase environmental sustainability. We assumed hourly dynamic prices, but the potential for responsive charging strategies will decrease in case of less dynamic time-of-use tariffs (e.g., day and night differentiation). Implementing smart charging requires available signals, ideally transmitted through smart metering, to enable dynamic pricing or real-time emission information and optimal charging strategies that minimize environmental impacts or electric system costs. In Switzerland, the differences between optimizing for costs and emissions are substantial, with price-responsive charging resulting in an average savings of 27% compared to emission-responsive charging, which could save, on average, 82% of emissions. However, the CO_2_ footprint of mobility is not only altered by shifting from a combustion to an electric motor and smart charging strategies but also by changing the car ownership model. Shared vehicles are used more frequently and thus show less temporal flexibility for charging during the daytime, requiring more overnight charging. Even though carsharing can take less advantage of hours with low CO_2_ emissions, overall, the aggregated differences to privately owned cars in CO_2_ emissions or costs are minimal. There would be no need to differentiate between smart charging strategies if the lowest electricity prices were perfectly aligned with the lowest emissions. Therefore, if the energy system is moving toward this direction, the smaller the difference will be between emission- and cost-responsive charging. In the future, a better alignment of low costs and low emissions can be expected through the large-scale deployment of solar in Switzerland and in neighboring countries. However, despite Europe’s path toward net-zero, spatial spillovers of high-carbon emissions and low prices may still occur among European countries as long as the phase-out of fossil-fuelled electricity generation, which can be used for balancing purposes, is left solely to market forces and not better coordinated at EU-level.^65^

To align private cost minimization with socially optimal emissions outcomes in EV charging, the results of this study indicate the need for explicit CO_2_ pricing or equivalent incentives by electricity tariffs. The simulated difference between cost- and emissions-optimized charging suggests that, under current market conditions, private owners and carsharing operators have limited financial motivation to charge during low-carbon hours. To internalize the emission externality and to make emission-optimal charging economically rational, an average carbon price of EUR 0.3/kg CO_2_-equivalent would be required (calculated from the differences of the average calculations from Table 2). This highlights the potential role of dynamic carbon tariffs or time-varying incentives that reflect real marginal emission intensities of the power system. Complementary measures may be necessary to fully leverage the emission-reduction potential for carsharing operators, such as facilitating fast charging during periods of low emissions.

The deployment of BEVs may differ by country, but the careful integration of renewables^66^ and decarbonization of the energy system^67^ are essential to minimize emissions and to consider the security of electricity supply. Carsharing is assumed to reduce the number of cars and energy consumption: Through an assumed reduction of 25% in absolute vehicle numbers in urban areas via carsharing (and a corresponding energy reduction of 25%), the security of electricity supply during winter months is simulated to be improved by 0.9 TWh for price-responsive and 1.1 TWh for emission-responsive charging by 2050 (see differences in total unsupplied electricity demand in Figure 7). Whereas the focus of this study was on changes in electricity demand from electrification of transport and carsharing, electrification needs to go in hand with electricity supply considerations in Switzerland and neighboring countries. Furthermore, the supporting physical and socio-technical infrastructure, both for electrification (e.g., charging stations at home or work, fiscal policies on BEV purchases, time-of-use tariffs) and carsharing (e.g., technologies and incentives for promoting carsharing, mobility-as-a-service, business models for carsharing) will need to fully mature and innovate to reap environmental and economic benefits at the system scale. For Switzerland, large-scale electrification of transport will require adjustments to the energy system to meet increased electricity demand from BEVs in winter. Although carsharing and emission-responsive charging may reduce necessary energy system adjustments, additional electricity demand in winter remains considerable, with a monthly maximum of 1 TWh. Whereas the numbers presented here are derived from the scenario in this work, the future energy-saving potential from carsharing or developments in the electricity supply sector will impact these figures. Finally, policymakers should not promote transport electrification through incentives such as tariffs or remuneration^55^ without considering smart meter deployment and providing both price and emission signals, while promoting carsharing or other modes of transport to shift toward more sustainable modes of transportation. Policymakers could use findings such as those presented here to justify support measures for carsharing during winter months. Furthermore, since private BEVs have more flexibility to shift charging to low-emission hours but only show minimal system-level efficiency gains compared to carsharing, incentive schemes could be rebalanced to provide greater support for shared fleets, especially if their charging behavior is optimized to reduce emissions.

## Supplementary Material



## Data Availability

The raw data is not publicly available because it contains commercially sensitive information. The data supporting this study’s findings are available upon request from the authors.

## Notes

Additional simulation results are provided that explore the sensitivity of the probability of charging for different modeling assumptions, namely the charging rate (Figure S1) and the distance traveled (Figure S2) (PDF)

## References

[1] Guéret A., Schill W.-P., Gaete-Morales C. (2024). Impacts of
Electric Carsharing on
a Power Sector with Variable Renewables. Cell
Rep. Sustainability.

[2] Amatuni L., Ottelin J., Steubing B., Mogollón J. M. (2020). Does Car
Sharing Reduce Greenhouse Gas Emissions? Assessing the Modal Shift
and Lifetime Shift Rebound Effects from a Life Cycle Perspective. J. Cleaner Prod..

[3] Wang W., Zhang Q., Peng Z., Shao Z., Li X. (2020). An Empirical
Evaluation of Different Usage Pattern between Car-Sharing Battery
Electric Vehicles and Private Ones. Transp.
Res. Part A: Policy Pract..

[4] Hoerler R., van Dijk J., Patt A., Del Duce A. (2021). Carsharing
Experience
Fostering Sustainable Car Purchasing? Investigating Car Size and Powertrain
Choice. Transp. Res. Part D: Transp. Environ..

[5] Manville M. (2017). Travel and
the Built Environment: Time for Change. J. Am.
Plann. Assoc..

[6] Loose, W. The State of European Car-Sharing: Final Report; Bundesverband CarSharing, 2010 p129.

[7] Gross, R. ; Heptonstall, P. ; Anable, J. ; Greenacre, P. What Policies Are Effective at Reducing Carbon Emissions from Surface Passenger Transport? A Review of Interventions to Encourage Behavioural and Technological Change; UKERC, 2009 p192.

[8] Morfeldt J., Johansson D. J. A. (2022). Impacts
of Shared Mobility on Vehicle Lifetimes and
on the Carbon Footprint of Electric Vehicles. Nat. Commun..

[9] Becker H., Balac M., Ciari F., Axhausen K. W. (2020). Assessing
the Welfare
Impacts of Shared Mobility and Mobility as a Service (MaaS). Transp. Res. Part A: Policy Pract..

[10] Rüdisüli M., Romano E., Eggimann S., Patel M. (2022). Decarbonization Strategies
for Switzerland Considering Embedded Greenhouse Gas Emissions in Electricity
Imports. Energy Policy.

[11] de
Sa A. L. S., Lavieri P. S., Cheng Y. T., Hajhashemi E., Oliveira G. J. M. (2023). Modelling Driver’s Response to Demand Management
Strategies for Electric Vehicle Charging in Australia. Energy Res. Soc. Sci..

[12] Bauer C., Hofer J., Althaus H. J., Del Duce A., Simons A. (2015). The Environmental
Performance of Current and Future Passenger Vehicles: Life Cycle Assessment
Based on a Novel Scenario Analysis Framework. Appl. Energy.

[13] Nordelöf A., Messagie M., Tillman A. M., Ljunggren Söderman M., Van Mierlo J. (2014). Environmental
Impacts of Hybrid, Plug-in Hybrid, and
Battery Electric Vehicles—What Can We Learn from Life Cycle
Assessment?. Int. J. Life Cycle Assess..

[14] Chakraborty D., Hardman S., Tal G. (2020). Why Do Some Consumers Not Charge
Their Plug-in Hybrid Vehicles? Evidence from Californian Plug-in Hybrid
Owners. Environ. Res. Lett..

[15] Choudhari T. P., Illahi U., Al-Hosni M., Caulfield B., O’Mahony M. (2024). Decarbonising Shared Mobility: The
Potential of Shared
Electric Vehicles. Transp. Res. Part D: Transp.
Environ..

[16] Yuksel T., Tamayao M. A. M., Hendrickson C., Azevedo I. M. L., Michalek J. J. (2016). Effect
of Regional Grid Mix, Driving Patterns and Climate on the Comparative
Carbon Footprint of Gasoline and Plug-in Electric Vehicles in the
United States. Environ. Res. Lett..

[17] Nunes A., Woodley L., Rossetti P. (2022). Re-Thinking
Procurement Incentives
for Electric Vehicles to Achieve Net-Zero Emissions. Nat. Sustainability.

[18] Li Y., Davis C., Lukszo Z., Weijnen M. (2016). Electric Vehicle Charging
in China’s Power System: Energy, Economic and Environmental
Trade-Offs and Policy Implications. Appl. Energy.

[19] Jenn, A. ; Brown, A. Green Charging of Electric Vehicles Under a Net-Zero Emissions Policy Transition in California, University of California, Davis, 2021.

[20] Jochem P., Babrowski S., Fichtner W. (2015). Assessing CO2 Emissions of Electric
Vehicles in Germany in 2030. Transp. Res. Part
A: Policy Pract..

[21] Luh S., Kannan R., McKenna R., Schmidt T. J., Kober T. (2023). How, Where,
and When to Charge Electric Vehicles – Net-Zero Energy System
Implications and Policy Recommendations. Environ.
Res. Commun..

[22] Kang Z., Ye Z., Lam C., Hsu S. (2023). Sustainable
Electric Vehicle Charging
Coordination : Balancing CO 2 Emission Reduction and Peak Power
Demand Shaving. Appl. Energy.

[23] Yang S. (2019). Price-Responsive
Early Charging Control Based on Data Mining for Electric Vehicle Online
Scheduling. Electr. Power Syst. Res..

[24] Matisoff D. C., Beppler R., Chan G., Carley S. (2020). A Review of Barriers
in Implementing Dynamic Electricity Pricing to Achieve Cost-Causality. Environ. Res. Lett..

[25] Léautier T.-O. (2014). Is Mandating
“Smart Meters” Smart?. Energy
J..

[26] Tang Y., Cockerill T. T., Pimm A. J., Yuan X. (2021). Reducing the Life Cycle
Environmental Impact of Electric Vehicles through Emissions-Responsive
Charging. iScience.

[27] Kenngrössen Zur Entwicklung Der Treibhausgasemissionen in Der Schweiz 1990–2021; Bundesamt für Umwelt, 2023 p72.

[28] IEAHEV . Switzerland EV Adoption by Year. https://ieahev.org/countries/Switzerland/. (accessed December 22, 2023).

[29] GoGreen . Entwicklung der Marktanteile alternative Antriebe. https://archive.is/rbCp5. (accessed May 25, 2025).

[30] Energy Strategy 2050 Monitoring Report; Swiss Federal Office of Energy: Ittigen, Switzerland; 2023.

[31] Mobilitätsverhalten Der Bevölkerung. Ergebnisse Des Mikrozensus Mobilität Verkehr 2021, Bundesamt für Statistik, 2023.

[32] Becker H., Ciari F., Axhausen K. W. (2017). Comparing
Car-Sharing Schemes in
Switzerland: User Groups and Usage Patterns. Transp. Res. Part A: Policy Pract..

[33] Mobility . Mobility Carsharing. https://www.mobility.ch/. (accessed April 17, 2024).

[34] Ben-Dor G., Ogulenko A., Klein I., Ben-Elia E., Benenson I. (2024). Simulation-Based
Policy Evaluation of Monetary Car Driving Disincentives in Jerusalem. Transp. Res. Part A: Policy Pract..

[35] Tajaddini, A. ; Rose, G. ; M Kockelman, K. ; L Vu, H. Recent Progress in Activity-Based Travel Demand Modeling: Rising Data and Applicability. In Models and Technologies for Smart, Sustainable and Safe Transportation Systems; IntechOpen, 2021 10.5772/intechopen.93827.

[36] Parajeles Herrera, M. ; Schwarz, M. ; Hug, G. Spatio-Temporal Modeling of Large-Scale BEV Fleets’ Charging Energy Needs and Flexibility; International Conference on Smart Energy Systems and Technologies (SEST); IEEE, 2024; pp 1–6.

[37] Horocarbon . Mon Electricité Est-elle Vraiment Verte ?. https://horocarbon.ch. (accessed July 11, 2024).

[38] Romano, E. ; Hollmuller, P. ; Patel, M. Émissions Horaires de Gaz à Effet de Serre Liées à La Consommation d’électricité – Une Approche Incrémentale Pour Une Économie Ouverte : Le Cas de La Suisse; Genève, 2018 p25.

[39] EEX Group . epexspot. https://www.epexspot.com/en. (accessed July 10, 2025).

[40] Sripad S., Viswanathan V. (2017). Performance Metrics Required of Next-Generation Batteries
to Make a Practical Electric Semi Truck. ACS
Energy Lett..

[41] Han D., Zamee M. A., Choi G., Kim T., Won D. (2024). Optimal Electrical
Vehicle Charging Planning and Routing Using Real-Time Trained Energy
Prediction with Physics-Based Powertrain Model. IEEE Access.

[42] Federal Statistical Office . Passenger Transport Performance. https://archive.is/wip/PrNhi. (accessed May 30, 2024).

[43] Sakkas, A. Simulation and Analysis of the Performance of a Traffic Network in Zurich; ETH Zurich, 2022.

[44] Apostolaki-Iosifidou E., Codani P., Kempton W. (2017). Measurement of Power Loss during
Electric Vehicle Charging and Discharging. Energy.

[45] Reick B., Konzept A., Kaufmann A., Stetter R., Engelmann D. (2021). Influence
of Charging Losses on Energy Consumption and CO2 Emissions of Battery-Electric
Vehicles. Vehicles.

[46] Energieperspektiven 2050+: Technischer Bericht Gesamtdokumentation Der Arbeiten; BFE: Bern; 2021.

[47] Doquet, M. ; Gonzalez, R. ; Lepy, S. ; Momot, E. ; Verrier, F. A New Tool for Adequacy Reporting of Electric Systems : ANTARES; CIGRE: Paris; 2008.

[48] ENTSO-E . TYNDP 2020 - Scenario Report, 2020. https://2020.entsos-tyndp-scenarios.eu/download-data/. (accessed April 17, 2024).

[49] VSE . Energiezukunft2050: Energieversorgung Der Schweiz Bis 2050, 2022. https://www.strom.ch/de/energiezukunft-2050/startseite. (accessed April 17, 2024).

[50] Moody J., Farr E., Papagelis M., Keith D. R. (2021). The Value of Car
Ownership and Use in the United States. Nat.
Sustainability.

[51] Bardwell L., Blackhall L., Shaw M. (2023). Emissions and Prices
Are Anticorrelated
in Australia’s Electricity Grid, Undermining the Potential
of Energy Storage to Support Decarbonisation. Energy Policy.

[52] Rupp M., Rieke C., Handschuh N., Kuperjans I. (2020). Economic and
Ecological Optimization of Electric Bus Charging Considering Variable
Electricity Prices and CO2eq Intensities. Transp.
Res. Part D: Transp. Environ..

[53] Sheppard C. J. R., Gopal A. R., Harris A., Jacobson A. (2016). Cost-Effective
Electric
Vehicle Charging Infrastructure Siting for Delhi. Environ. Res. Lett..

[54] Guéret A., Schill W.-P., Gaete-Morales C. (2024). Impacts of
Electric Carsharing on
a Power Sector with Variable Renewables. Cell
Rep. Sustainability.

[55] Syla A., Rinaldi A., Parra D., Patel M. K. (2024). Optimal Capacity
Planning for the Electrification of Personal Transport : The
Interplay between Flexible Charging and Energy System Infrastructure. Renewable Sustainable Energy Rev..

[56] Geels F. W. (2012). A Socio-Technical
Analysis of Low-Carbon Transitions: Introducing the Multi-Level Perspective
into Transport Studies. J. Transp. Geogr..

[57] Anable J., Brand C., Tran M., Eyre N. (2012). Modelling Transport
Energy Demand: A Socio-Technical Approach. Energy
Policy.

[58] Kemp R., Schot J., Hoogma R. (1998). Regime Shifts to Sustainability through
Processes of Niche Formation: The Approach of Strategic Niche Management. Technol. Anal. Strategic. Manage..

[59] Kemp R., Schot J., Hoogma R. (2007). Regime Shifts to Sustainability
through
Processes of Niche Formation : The Approach of Strategic Niche
Management Regime Shifts to Sustainability Through Processes of Niche
Formation : The Approach of Strategic Niche Management. Technol. Anal. Strategic Manage..

[60] Fitschen P., Merfeld K., Klein J. F., Henkel S. (2024). Understanding the Urban
Mobility Challenge: Why Shared Mobility Providers Fail to Attract
Car Drivers. Transp. Policy.

[61] Burghard U., Dütschke E. (2019). Who Wants Shared Mobility? Lessons
from Early Adopters
and Mainstream Drivers on Electric Carsharing in Germany. Transp. Res. Part D: Transp. Environ..

[62] Illgen S., Höck M. (2018). Electric Vehicles in Car Sharing
Networks –
Challenges and Simulation Model Analysis. Transp.
Res. Part D: Transp. Environ..

[63] Brendel A. B., Lichtenberg S., Brauer B., Nastjuk I., Kolbe L. M. (2018). Improving
Electric Vehicle Utilization in Carsharing: A Framework and Simulation
of an e-Carsharing Vehicle Utilization Management System. Transp. Res. Part D: Transp. Environ..

[64] Logan K. G., Nelson J. D., Brand C., Hastings A. (2021). Phasing in
Electric
Vehicles: Does Policy Focusing on Operating Emission Achieve Net Zero
Emissions Reduction Objectives?. Transp. Res.
Part A: Policy Pract..

[65] Romano E., Mutschler R., Hollmuller P., Sulzer M., Orehounig K., Rüdisüli M. (2024). Spatial Carbon
and Price Spillovers
among EU Countries on Their Pathway toward Net-Zero Electricity Supply. Energy Econ..

[66] Ayetor G. K. (2022). Towards
Net Zero Electric Vehicle Emissions in Africa. Curr. Sustainable Energy Rep..

[67] Leard B., Greene D. (2023). Coordinating the Electric
Vehicle Transition and Electricity
Grid Decarbonization in the U.S. Is Not Essential to Achieving Substantial
Long-Term Carbon Dioxide Emissions Reductions. Environ. Res. Lett..

