# Correction to “Emission-Responsive Charging of Electric Cars and Carsharing to Improve the Security of Electricity Supply for Switzerland”

**DOI:** 10.1021/acs.est.5c18347

**Published:** 2026-01-30

**Authors:** Elliot Romano, Binod Koirala, Martin Rüdisüli, Sven Eggimann

The reported 27% savings of price-responsive charging in the abstract and section 4.2 was incorrect, with the correct value being 21%. Also, in section 3.4, the savings of CO_2_ emissions for emission-responsive charging is 82% and not 81.4% (as correctly reported in Table 1). In the labeling of Figure 3, the right axis is shown with a red line and the left axis with a blue line, consistent with the graphical legend. Apart from these corrections in the reported numbers, none of the conclusions or simulations are affected.

