# Supplementary material for: Emission-Responsive Charging of Electric Cars and Carsharing to Improve the Security of Electricity Supply for Switzerland
Source: Environ Sci Technol. 2025 Jul 17;59(29):15057–69. doi: 10.1021/acs.est.4c13270 (PMC12312090; doi:10.1021/acs.est.4c13270)
Supplement: Supplementary file 1 [file es4c13270_si_001.pdf]

Supporting Information for:

Emission-responsive charging of electric cars and carsharing to improve the security of electricity supply for Switzerland

Elliot Romano†[1,2], Binod Koirala[2], Martin Rüdisüli[3], Sven Eggimann†[4]*

1 Institute for Environmental Sciences and Department F.-A. Forel for Environmental and Aquatic Sciences, Energy Systems Group, University of Geneva, 1205 Geneva, Switzerland

2 Urban Energy Systems Laboratory, Swiss Federal Laboratories for Materials Science and Technology, Empa, CH-8600 Dübendorf, Switzerland

3 Verband Schweizerischer Elektrizitätsunternehmen, CH-5000 Aarau, Switzerland

4 Department of Geography, The Hebrew University of Jerusalem, Mount Scopus, Jerusalem 91905, Israel

* corresponding author, †co-first authors

Summary:

Number of pages	3
Number of figures	2

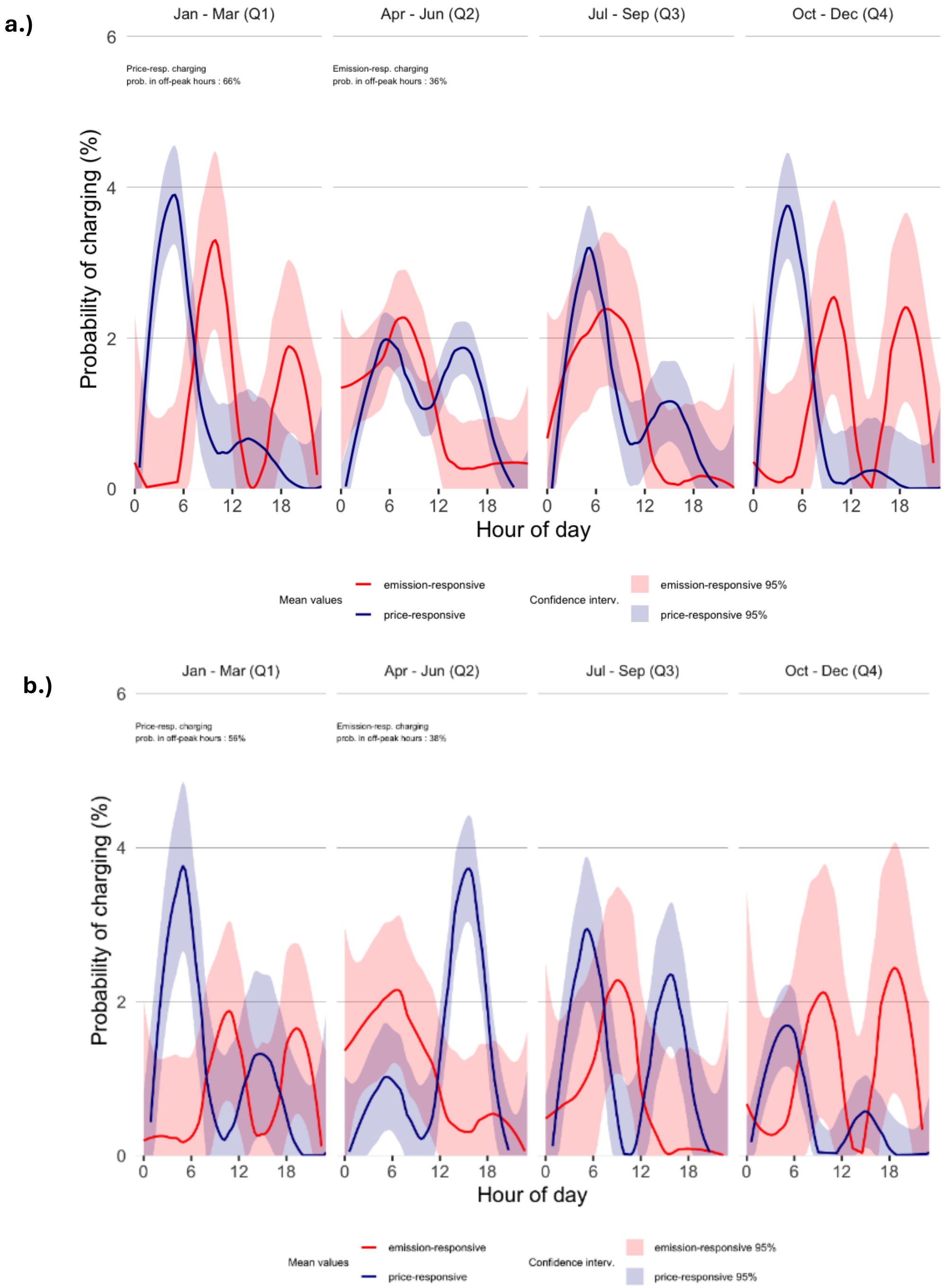

Figure S1: Sensitivity of probability of charging for different charging rate assumptions. The simulated probability of charge for a.) regular charging (11kW) and b.) very fast charging (55 kW) are shown.

a.)

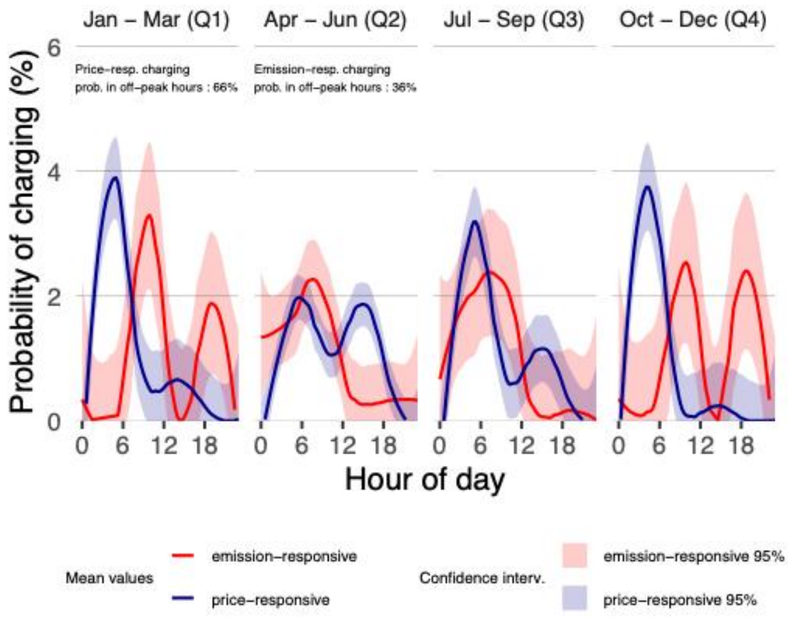

b.)

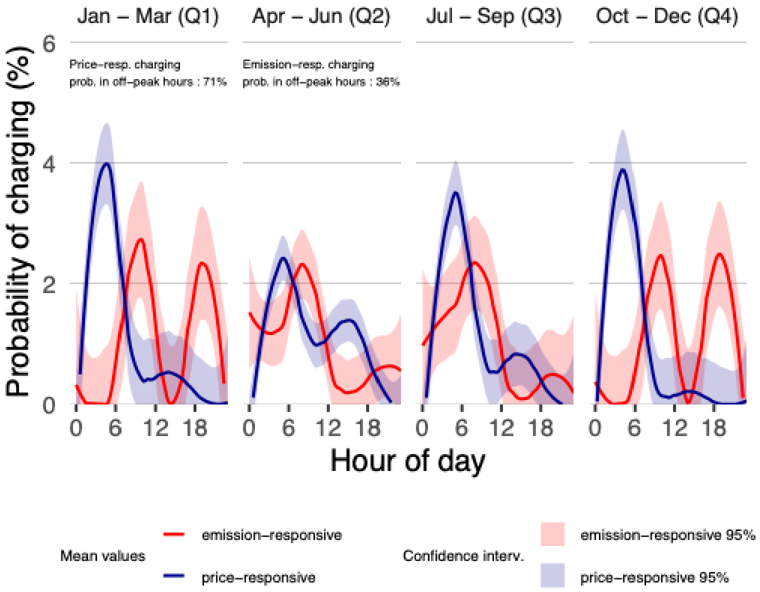

Figure S2: Sensitivity of probability of charging for a.) assumed same distance travelled for the individual and shared mobility scheme and b.) for assumed double distance travelled for the shared mobility scheme.
